# Investigation of the Effect of Pallet Top-Deck Stiffness on Corrugated Box Compression Strength as a Function of Multiple Unit Load Design Variables

**DOI:** 10.3390/ma14216613

**Published:** 2021-11-03

**Authors:** Saewhan Kim, Laszlo Horvath, Jennifer D. Russell, Jonghun Park

**Affiliations:** 1 Department of Sustainable Biomaterials, Virginia Polytechnic Institute and State University, 1650 Research Center Drive, Blacksburg, VA 24061, USA; seabed94@vt.edu (S.K.); jdrussell@vt.edu (J.D.R.); 2 School of Graphic Communications Management, Ryerson University, 125 Bond Street, Toronto, ON M5B 2K3, Canada; jaypark@ryerson.ca

**Keywords:** corrugated box, compression strength, pallet, unit load, unit load optimization

## Abstract

Unit loads consisting of a pallet, packages, and a product securement system are the dominant way of shipping products across the United States. The most common packaging types used in unit loads are corrugated boxes. Due to the great stresses created during unit load stacking, accurately predicting the compression strength of corrugated boxes is critical to preventing unit load failure. Although many variables affect the compression strength of corrugated boxes, recently, it was found that changing the pallet’s top deck stiffness can significantly affect compression strength. However, there is still a lack of understanding of how these different factors influence this phenomenon. This study investigated the effect of pallet’s top-deck stiffness on corrugated box compression strength as a function of initial top deck thickness, pallet wood species, box size, and board grade. The amount of increase in top deck thickness needed to lower the board grade of corrugated boxes by one level from the initial unit load scenario was determined using PDS™. The benefits of increasing top deck thickness diminish as the initial top deck thickness increases due to less severe pallet deflection from the start. The benefits were more pronounced as higher board grade boxes were initially used, and as smaller-sized boxes were used due to the heavier weights of these unit loads. Therefore, supposing that a company uses lower stiffness pallets or heavy corrugated boxes for their unit loads, this study suggests that they will find more opportunities to optimize their unit loads by increasing their pallet’s top deck thickness.

## 1. Introduction

Historically, the distribution packaging industry has adapted the method of unitizing single, multiple, or bulk products on a solid platform to make the handling, storing, and transporting of these products easier [1]. This arrangement is called a unit load. In today’s supply chains, 80% of products are moved in unit load form [2]. The most common base platform for unit loads is a pallet. Pallets can be made of different materials such as wood, plastic, paper, or metal. Among these materials, wood is by far the most commonly used to manufacture pallets. Wood is the material of choice for over 90% of companies that use pallets in their supply chains in the United States [3]. Furthermore, approximately 804 million new and recycled wood pallets were manufactured in 2016 [4]. Just as wood pallets have become one of the essential elements of a unit load, corrugated boxes also play a crucial role. Corrugated boxes are the most used primary and secondary packaging; in fact, 72% of unit loads are built using corrugated boxes [3].

When designing a unit load, accurately predicting corrugated box compression strength is crucial to avoid package failure from the vertical compression forces during distribution and storage. Therefore, numerous studies have investigated the factors that affect the compression strength of corrugated boxes, including material properties [5,6,7,8,9,10], manufacturing methods [6,11,12,13,14,15,16], environmental condition factors [6,8,12,17], and the palletization factor [18,19,20,21,22,23,24,25,26].

Wood pallet characteristics, such as pallet gaps and pallet overhang, have been included among the main palletization factors that affect box compression strength. In relatively recent years, researchers have endeavored to correlate pallet top-deck stiffness to corrugated box compression strength. Baker [19] and Phanthanousy [24] examined the relationships between the differences in stress concentrations and box compression strength. However, their studies were inconclusive. Phanthanousy found that the stiffness of the pallet’s top deck has no notable effect on box compression strength when the wood pallet is designed with deck board gaps.

Meanwhile, Baker [19] found that pallet top-deck stiffness significantly affects box compression strength when the wood pallet is designed with no deck board gaps. Their studies only evaluated situations in which all corners of the boxes were symmetrically supported. However, in many cases, the top deck board of a wood pallet deforms by the weight of the top load and creates asymmetric support conditions for the loaded products. Baker [19] highlighted that asymmetrically supported corrugated boxes are a prevalent condition in most unit loads, and his research found that asymmetric support can decrease corrugated box compression strength by as much as 15%.

In 2020, Quesenberry et al. [25] further investigated the effect of wood pallet top-deck stiffness on corrugated box compression strength when box corners are asymmetrically supported. They concluded that a stiffer top deck board could increase the compression strength of asymmetrically supported corrugated boxes up to 37% when the unit loads are double-stacked on the floor [25]. They also discovered that the effect of pallet top-deck stiffness on box compression strength could be utilized to lower the cost of a unit load by decreasing the required board grade of corrugated boxes and increasing the pallet’s top deck thickness. However, the experimental design utilized by Quesenberry only focused on a limited number of variables. Furthermore, the pallet design utilized for his experimental unit load consisted of a single wood species and singular moisture content.

Additionally, the corrugated boxes were made of a single board grade, two flute sizes, and two box sizes. In practice, many wood species with varying moisture content are available for pallet manufacturing; meanwhile, corrugated boxes are produced in multiple board grades and sizes. Nevertheless, there is an absence of studies investigating how these variations may change the effect of top deck stiffness on corrugated box compression strength.

Therefore, the objective of this current paper is to investigate the effect of pallet top-deck stiffness on the compression strength of asymmetrically supported corrugated boxes as a function of currently under-studied variables, including initial top deck thickness, pallet wood species, box size, and board grade.

## 2. Materials and Methods

This study consisted of two main sections: validation of the analytical pallet design software and unit load scenario analysis.

### 2.1. Software Validation

The commercially available pallet design software Pallet Design System™ (PDS™) v. 6.2, created by NWPCA (National Wooden Pallet & Container Association, Alexandria, VA, USA) was utilized to replace numerous physical experiments in this study. The box performance data predicted by PDS™ and that Quesenberry et al. [25] found were compared to confirm that the software reproduced the results from the experiment.

#### 2.1.1. Corrugated Box Description for Validation

The same designs of corrugated boxes used by Quesenberry et al. [25] were used to build the unit load model in PDS™ for predictive software validation. Specified parameters from Quesenberry et al. [25] included: Regular Slotted Container (RSC) style with two different external dimensions (length × width × height) 406.4 mm × 247.7 mm × 254 mm and 609.6 mm × 247.7 mm × 254 mm. Unit loads were built with four layers of boxes, and the configuration of boxes was either 3 boxes × 4 boxes (length × width) or 2 boxes × 4 boxes. Both sizes of boxes were built with nominal 0.57 kg/mm Edge Crush Test (ECT) value B-flute and C-flute corrugated board.

#### 2.1.2. Pallet Description for Validation

**Figure 1:**
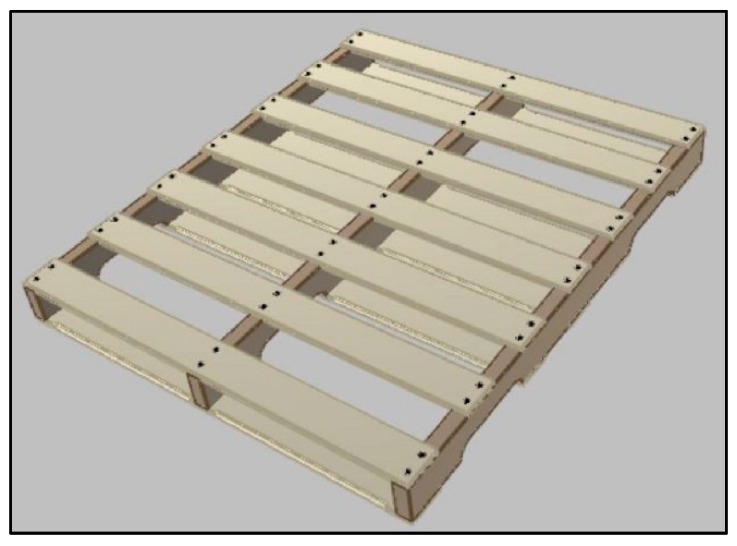
Picture of GMA pallet used for software validation (image generated using PDS™).

Quesenberry et al. [25] simulated a 1219.2 mm × 1016 mm GMA™ (Grocery Manufacturers Association) style pallet by using a custom-built, quarter-section pallet for testing purposes. For software validation, a full-sized 1219.2 mm × 1016 mm stringer class, double face, non-reversible, partial four-way, unidirectional bottom, flush, GMA™ style pallet was modeled in PDS™ (see Figure 1). The pallet consisted of three stringers, seven top deck boards, five bottom deck boards, and two fasteners per joint. The stringers were 1219.2 mm long, 31.8 mm wide, and 88.9 mm high. The top and bottom deck boards were 1016 mm long and 88.9 mm wide. The four top deck board thicknesses studied were: 9.5 mm, 12.7 mm, 15.9 mm, and 19.1 mm. All top deck boards were equally spaced 99.6 mm apart. Lead bottom deck boards were spaced 292.1 mm away from the interior bottom deck boards, and the interior bottom deck boards were spaced 95.3 mm apart. Number 1 & better (premium & better), kiln-dried, Spruce–Pine–Fir (SPF) lumber was used for all pallet components.

#### 2.1.3. Comparison of Box Load Factor and Box Compression Strength Factor

During software validation, the box load factors computed by PDS™ and the box compression strength factor derived from the thesis of Quesenberry [27] were compared. The box load factor is the ratio of the weights on worst loaded box edges to the load if it were evenly distributed. Meanwhile, the box compression strength factor is a new term developed by the authors and is defined as the ratio of the box compression strength when box corners are symmetrically supported on rigid supports to the box compression strength when its corners are asymmetrically supported on an actual pallet. Both the box load factor and the box compression strength factor ultimately provide information about the compression performance of the corrugated box.

##### Process of Computing Box Load Factor

Box load factors were computed using PDS™ following the steps described below.

*Step 1:* Built a unit load in PDS™ using boxes and pallets previously described in Section 2.1.1 and Section 2.1.2.
*Step 2:* Set the top deck board thickness to the lowest level (9.5 mm).
*Step 3:* Set the weight in the box to the load that will just fail the boxes (box safety factor of one) when the support condition is a single floor stack.
*Step 4*: Report current box load factor when support condition is single floor stack.
*Step 5*: Increased the top deck board thickness to the following levels (12.7 mm, 15.9 mm, and 19.1 mm).
*Step 6*: Repeat *steps 3* and *4* for each level of top deck board thickness.
*Step 7:* Repeat the process for two flute sizes (B and C flute) and two box sizes.

##### Process of Calculating Box Compression Strength Factor

The box compression strength factor from Quesenberry’s study was calculated using Equation (1):

(1) $$CSF=\frac{SCS_{avg}}{ACS_{avg}}$$

where:

*CSF* = Box compression strength factor.
*SCS_avg_* = Average box compression strength when box corners are symmetrically supported on a rigid platform.
*ACS_avg_* = Average box compression strength when box corners are asymmetrically supported on the actual pallet.
The unit load scenarios used to calculate the box compression strength factors were varied by two flute sizes, two box sizes, and four thickness levels.

##### Statistical Analysis

The independent *t*-test was conducted to see whether the difference between box load factors from PDS™ and box compression strength factors from the experiment were statistically significant or not. To confirm the normality assumption of the independent *t*-test, we also ran the Shapiro–Wilk test for each group separately. The similarities between the box performance data from PDS™ and the experiment were also assessed using the Pearson correlation coefficient. The Pearson correlation coefficient is a way to investigate linear dependence between two variables. The measured correlation coefficient (*r*) ranges between −1 and +1. When the *r*-value is −1, it indicates a strong negative correlation, while +1 indicates a strong positive correlation, and 0 means no relation. Both statistical analyses were conducted at a significance level of 0.05. The analyses were done using SAS JMP Pro 15^®^ software (SAS Enterprises, Raleigh, NC, USA).

### 2.2. Unit Load Scenario Analysis

The concept of a unit load cost optimization method that allows for corrugated boxes with decreased board grades by increasing the pallet’s top deck thickness was adopted from Quesenberry et al. [25] to modify each unit load scenario. In other words, the analysis was done by determining how much the top deck thickness needed to increase to lower the corrugated board grade by one level from the initial unit load scenario’s specific deck board thickness and board grade. A total of 234 unit load scenarios were designed with varying factors for investigation.

#### 2.2.1. Corrugated Box Description for Unit Load Scenario Analysis

**Figure 2:**
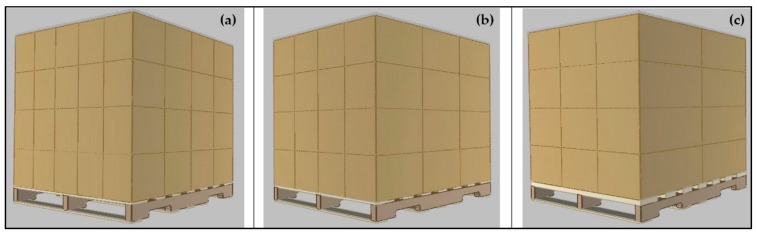
Image of investigated unit load configurations (image generated using PDS™). (**a**) Unit load with small boxes, (**b**) unit load with medium boxes, and (**c**) unit load with large boxes.

Three sizes of RSC-style corrugated boxes were investigated to explore the effect of different box sizes. Three box sizes were chosen that would cover the entire top surface of the 1219.2 mm × 1016 mm pallet and create asymmetrically supported corners. The external dimensions were 203.2 mm × 304.8 mm × 254 mm (small box), 406.4 mm × 254 mm × 254 mm (medium box), and 609.6 mm × 337.8 mm × 254 mm (large box). The boxes were organized in 4 × 5, 3 × 4, and 2 × 3 arrays for small, medium, and large boxes, respectively. Four layers of boxes were used for each unit load. Unit load configurations using the different box sizes are depicted in Figure 2. The boxes were built with two different flute sizes: single-wall C-flute and double-wall BC-flute. The C-flute and BC-flute corrugated boards were made of commonly manufactured board grades for each flute size. C-flute boards were modeled with nominal 0.52 kg/mm., 0.57 kg/mm, 0.71 kg/mm, and 0.79 kg/mm ECT. BC-flute boards were modeled with nominal 0.86 kg/mm, 0.91 kg/mm, 1.09 kg/mm, and 1.27 kg/mm ECT.

#### 2.2.2. Pallet Description for Unit Load Scenario Analysis

**Figure 3:**
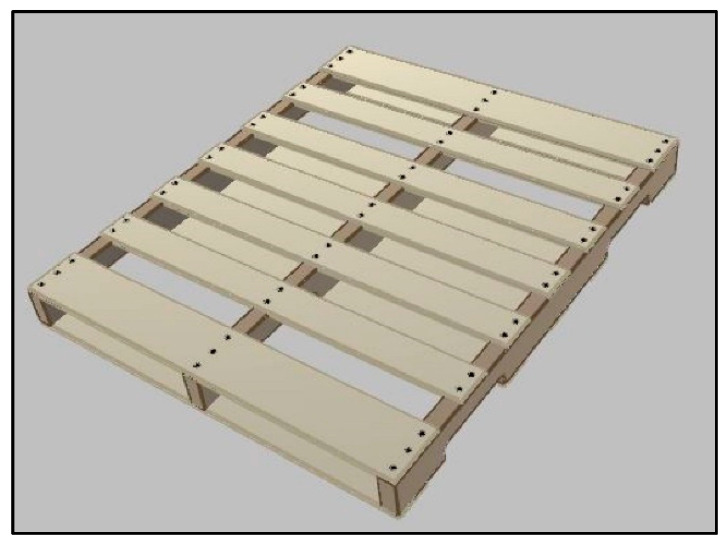
Picture of GMA pallet used for analysis (image generated using PDS™).

For the unit load scenario analysis, the most common size of GMA™ style wood pallet was used. The 1219.2 mm × 1016 mm GMA™ style stringer class wooden pallet is the most commonly used pallet design in North America [28,29]. The specifications were 1219.2 mm × 1016 mm, stringer class, double face, non-reversible, partial four-way, unidirectional bottom, flush, GMA™ style pallet (see Figure 3). The pallet design had three stringers, two lead top deck boards, five interior top deck boards, five bottom deck boards, two fasteners per joint on the interior top deck boards and for all bottom deck board connections, and three fasteners per joint on the lead top deck boards. The pallet design utilized for the unit load scenario analysis (Figure 3) had 50.8 mm wider lead top deck boards than the pallet design used for the software validation process (Figure 1). The spacing between top deck boards has also changed accordingly. The stringers were 1219.2 mm long, 31.8 mm wide, and 88.9 mm high. The interior top deck boards and bottom deck boards were 1016 mm long and 88.9 mm wide. The lead top deck boards were 1016 mm long and 139.7 mm wide. The bottom deck boards were 9.5 mm thick. All top deck boards were spaced 82.6 mm apart. The lead bottom deck boards were spaced 292.1 mm apart from interior bottom deck boards, and interior bottom deck boards were spaced 95.3 mm apart from each other. Number 1 & better (premium & better) grade lumber was used for all pallet components.

Initial top deck thicknesses were varied by four levels to explore which changes in deck board thicknesses would be required to reduce by one level the initial board grade specified for the corrugated boxes. The investigated initial top deck thickness levels were 9.5 mm, 12.7 mm, 15.9 mm, and 19.1 mm. However, unit load scenarios built with kiln-dried southern yellow pine (KD SYP) pallets were designed with 11.1 mm top deck boards, and this thickness was increased to 17.5 mm for the optimized design. This limitation was due to the availability of raw material sizes; only the 11.1 mm and 17.5 mm dimensions could be manufactured effectively.

Wood species used for pallet construction were also varied. The wood species commonly used for pallet construction in the southeastern United States were selected. Selected wood materials were: green, high-density hardwood (Grn HD HW); green, low-density hardwood (Grn LD HW); green, southern yellow pine (Grn SYP); and kiln-dried, southern yellow pine (KD SYP). Green lumber contained 25% or greater moisture content, and kiln-dried lumber had a maximum of 19% moisture content.

#### 2.2.3. Variable Factors

Several factors of the unit load were varied to identify the characteristics that could change the effect of the pallet’s top deck board stiffness on box compression strength. The factors evaluated were initial top deck board thickness, pallet wood species, box size, and corrugated board grade. The variable factors that relate to pallets are listed in Table 1, and Table 2 contains the variable factors relating to the boxes.

#### 2.2.4. Analysis Method

##### Measurement of Top Deck Thickness Increase

The unit load cost optimization method adopted from Quesenberry et al. [25] was investigated by varying the factors introduced in Section 2.2.3. The change in top deck thickness required to reduce by one level the corrugated board grade used, without downgrading box performance, was measured. This analysis was done with the unit load in the double floor stacked condition. A box safety factor of 3 was selected for the unit load design to comply with the requirements of the ISTA 3E testing standard [30].

Required steps in the analysis were as follows:

*Step 1:* Construct the unit load in PDS™.
*Step 2:* Set pallet material as one of the listed wood species (i.e., green high-density hardwood).
*Step 3:* Set the top deck board as the lowest initial top deck board thickness (9.5 mm). In the case of KD SYP, always set the initial top deck thickness as 11.1 mm.
*Step 4:* Set corrugated boxes as the higher ECT values in the selected range of board grade (i.e., Choose 0.57 kg/mm if the range was decreasing from 0.57 kg/mm to 0.52 kg/mm).
*Step 5:* Determine the weight of the box that works to create a box safety factor of three for the double floor stacking condition.
*Step 6:* Create a new unit load with the corrugated boxes made of lower ECT value from the selected range of corrugated board grade and apply the weight determined in *step 5* (i.e., Select 0.52 kg/mm if the range was 0.57 kg/mm to 0.52 kg/mm).
*Step 7:* Continuously increase the top deck thickness by 1.6 mm until the unit load again reaches the safety factor of three for safe operation. In the case of KD SYP, always increase the top deck thickness to 17.5 mm.
*Step 8:* Report the total increase in the top deck board thickness required to achieve the required safety factor of three.
*Step 9:* Repeat *step 1* to *step 8* after changing the pallet wood species.
*Step 10:* Repeat from *step 1* to *step 9* after increasing the initial top deck stiffness level.
*Step 11:* Repeat from *step 1* to *step 10* after changing the range of board grade (i.e., changing from a range of 0.57–0.52 kg/mm to a range of 0.71—0.57 kg/mm).
*Step 12:* Repeat from *step 1* to *step 11* after changing the size of corrugated boxes (i.e., changing from a small to a medium size box).

##### Unit Load Scenario Classification System

The amount that the top deck thickness increased was categorized as one of three grades to make it easier to identify which scenarios had smaller or larger increases in top deck thickness: less than 12.7 mm (grade 1), 12.7 mm to 25.4 mm (grade 2), and beyond 25.4 mm increase (grade 3). For better visualization, a color-coding system was also applied; green for grade 1, yellow for grade 2, and red for grade 3. Grade 1 scenarios were considered as cases with high potential to apply the unit load optimization process. Grade 2 scenarios were considered cases that may be possible to apply the optimization method depending on the manufacturer’s circumstances. Because pallets made of deck boards thicker than 25.4 mm are unprecedented; grade 3 scenarios were considered unrealistic unit load designs.

## 3. Results and Discussion

### 3.1. Software Validation Results

**Figure 4:**
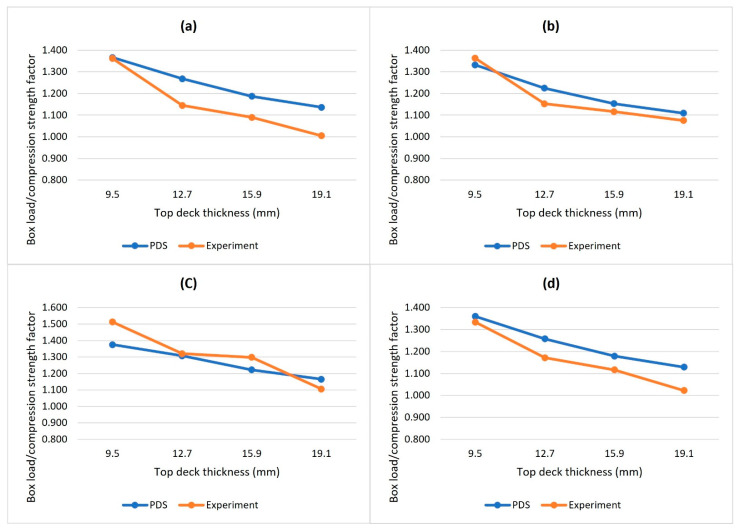
Comparison of box load factors and box compression strength factors of each type of boxes in response to pallet top-deck thickness. (**a**) Small C-flute box scenarios, (**b**) shows large C-flute box scenarios, (**c**) shows small B-flute box scenarios, and (**d**) shows large B-flute box scenarios.

Measurement of the box load factors and box compression strength factors on varied top deck thicknesses, box sizes, and flute sizes are presented in Table 3. The comparison of box load factors and box compression strength factors is plotted in Figure 4. It was observed that PDS™ tends to overestimate the effect of top deck stiffness when compared to the experiment results. However, the independent *t*-test showed that the difference between PDS™ and the experiment was not statistically significant (*t*_(25)_ = −0.85, *p*-value = 0.40). The Shapiro-Wilk test confirmed that the normality assumptions were met (PDS: W = 0.927, *p*-value = 0.216; Quesenberry: W = 0.919, *p*-value = 0.160). Furthermore, the Pearson correlation coefficient revealed a strong positive correlation, *r* = 0.911 (*p*-value < 0.0001), between box load factors from PDS™ and box compression strength factors from experiment results. In other words, the PDS™ and Quesenberry’s [27] experiments had a similar pattern.

### 3.2. Unit Load Scenario Analysis Results

Table 4 and Table 5 report the amount of top deck board thickness increase required to reduce the corrugated board grade by one level as a function of starting top deck thickness, wood species, initial board grade, and box sizes for the unit loads consisting of C-flute boxes and BC-flute boxes, respectively. A streamlined grading system has been applied, as described in Section 2.2.4, for better visualization and identification of the level of top deck thickness increase. The top deck thickness increase for grade 3 scenarios was reported as N/A (not applicable) because adding an extra inch of thickness to a pallet deck board is highly cost-prohibitive.

Table 6 and Table 7 present the KD SYP scenarios’ amount of top deck board thickness increase required to reduce the corrugated board grade by one level as a function of the different factors for the unit loads built using C-flute and BC-flute boxes, respectively.

To investigate how different factors such as the initial top deck board thickness, pallet wood species, box size, and board grade effect the feasibility of optimizing the strength of the corrugated boxes by changing the stiffness of the pallets, researchers looked at the changes in the proportions of different grade scenarios in response to each variable factor.

**Figure 5:**
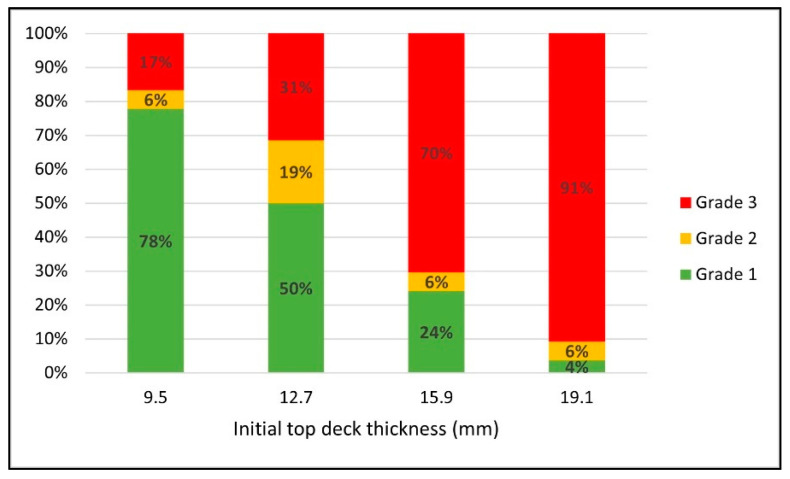
Changes in the proportions of the different grade scenarios in response to the initial top deck thickness for green wood scenarios.

Figure 5 shows how the proportions of various grade scenarios changed when different initial top deck thicknesses were used for the pallet design. As the initial top deck thickness increased, there was a significant reduction in the proportion of grade 1 scenarios. These are the scenarios where it is highly feasible to reduce the corrugated board grade with a reasonable amount of top deck thickness change. The proportion of grade 1 scenarios started from 78% with 9.5 mm initial top deck thickness and decreased to 50%, 24%, and 4% when the initial top deck thickness was 12.7 mm, 15.9 mm, and 19.1 mm, respectively. Correlatingly, the ratio of grade 3 scenarios was almost inversely proportional to the ratio of grade 1 scenarios as the initial top deck thickness increased. The proportion of grade 3 increased from 17% to 31%, 70%, and 91% when the initial top deck thickness was 9.5 mm, 12.7 mm, 15.9 mm, and 19.1 mm, respectively. Unlike other grade scenarios, no consistent trend was found in the proportion of grade 2 scenarios.

**Figure 6:**
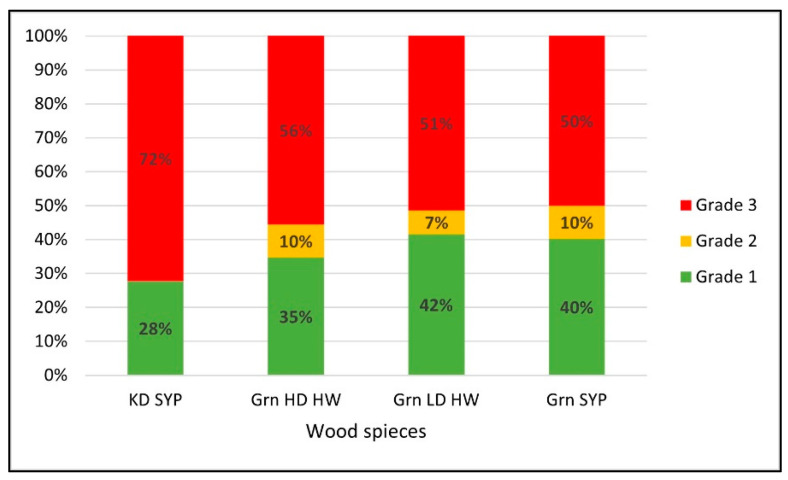
Changes in the proportions of the different grade scenarios in response to the pallet wood species. Note: KD SYP: kiln-dried southern yellow pine, Grn HD HW: green high-density hardwood, Grn LD HW: green low-density hardwood, Grn SYP: green southern yellow pine.

Figure 6 shows the changes in the proportions of the various unit load scenario grades when different wood species were used to build the pallets. The percent of different grade levels were similar for the scenarios using green low-density hardwood and green SYP with around 40% grade 1, 10% grade 2, and 50% grade 3. KD SYP scenarios behaved differently than the other wood species scenarios. They had a much lower number of feasible scenarios than the others. Grade 1 scenarios of KD SYP accounted for only 28%, while grade 1 scenarios of green lumber accounted for between 35–40%. The reduction of feasible scenarios might be attributable to the high stiffness of the KD SYP species. A highly stiff top deck will not bend enough to make a difference in board grade when top deck thickness changes. In addition, the results could have been affected by the limited availability of various KD SYP thicknesses. KD SYP lumber required a larger jump in top deck thicknesses than the 1.59 mm increases used with green species.

Furthermore, the proportion of the grade 1 scenarios for Grn HD HW was slightly lower (35%) than the other green lumber scenarios (40–42%). Since Grn HD HW does not have a limit on the level of top deck thickness increase, this could provide further evidence that the stiffness of the material affects the feasibility of the design scenario. Overall, the results indicate that the feasibility of using increased deck board thickness to lower the corrugated boxboard grade decreases when species with higher material stiffness are initially used to construct the pallets.

Similar trends in the proportional changes of different unit load scenario grades were observed from the initial top deck thickness effect and the pallet wood species effect. Both results indicated a significant reduction in the potential to decrease board grade by increasing top deck stiffness when the pallet was initially designed with stiffer pallet wood material. In other words, this unit load optimization method is more effective when the unit load is initially designed using lower stiffness pallets.

**Figure 7:**
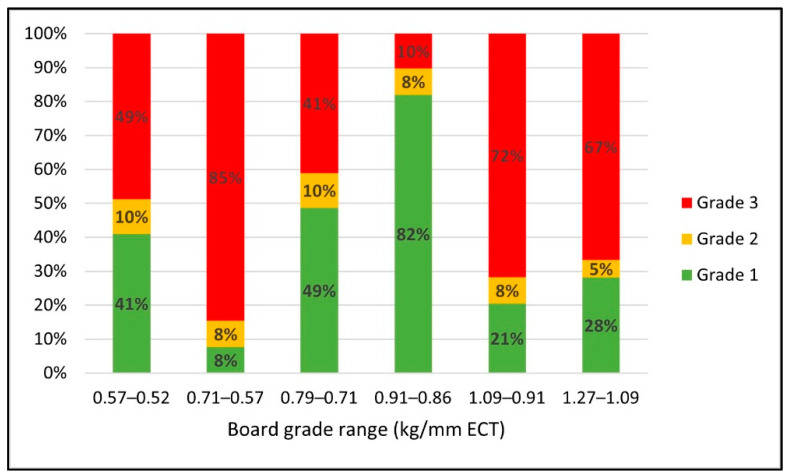
Changes in the proportions of the different grade scenarios in response to the range of board grade reduction.

Figure 7 displays changes in the proportions of different grade scenarios as a function of the range of board grade reduction. It was discovered that for the scenarios where the ECT change is greater between the consecutive board grade levels, the proportion of grade 1 scenarios decreases, and the ratio of grade 3 considerably increases. The ratio of grade 1 scenarios ranged between 41% and 82% for the cases with 0.05 kg/mm to 0.08 kg/mm ECT reduction. On the other hand, the proportion of grade 1 scenarios ranged only between 8% and 28% when it required 0.14 kg/mm to 0.18 kg/mm ECT value reduction. These results also show that the higher the initial board grade is, the more opportunities there are to reduce the board material with minor changes to top deck thickness. For instance, the proportion of grade 1 scenarios significantly increased from 41% to 49% and 82% when the board grade reduction range was 0.57–0.52 kg/mm, 0.79–0.71 kg/mm, and 0.91–0.86 kg/mm ECT, respectively. In this analysis, higher board grade also meant that the boxes supported more weight than lower board grade boxes. It indicates that the effect was more prominent for scenarios that had greater unit load weight because having more weight in the boxes causes more bending to the deck boards, which increases stress concentrations on the boxes.

**Figure 8:**
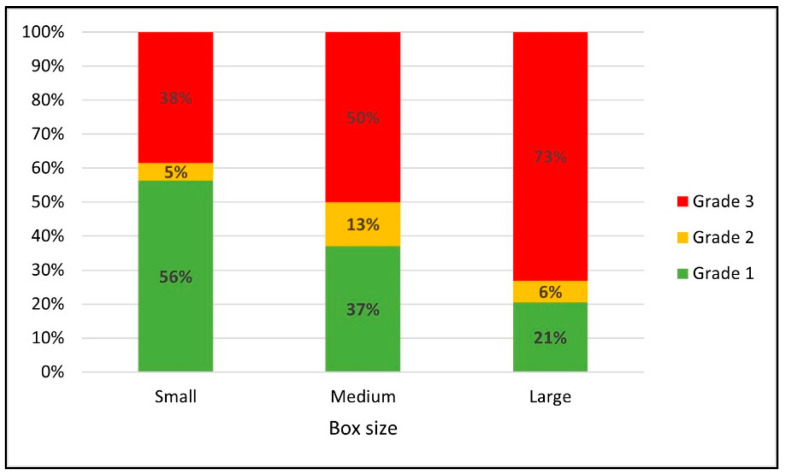
Changes in the proportions of the different grade scenarios in response to the box sizes.

Figure 8 shows changes in the proportions of various unit load scenario grades for the three different box sizes. The proportion of the grade 1 scenarios decreased from 57% to 39% and 21% and the proportion of the grade 3 scenario increased from 38% to 47% and 72% as package size increased from small to medium to large boxes. There was no consistent trend with the proportion of grade 2 scenarios. The results indicated that the feasibility is greater to reduce the corrugated board grade by increasing the thickness of top deck boards for unit loads consisting of small-sized boxes rather than larger ones. Similar to the board grade effect, this trend could be explained by weight differences per unit load. Although each small box held a lighter weight than the medium and large boxes in this analysis, the small box scenarios contained much heavier weight as a whole unit load than the scenarios with larger-sized boxes because these unit loads required more of the small boxes to create the same size load.

Overall, it was found that all investigated variable factors had an observable influence on the feasibility of using an increase in pallet top-deck stiffness to lower the board grade of the corrugated boxes. Unit load scenarios to which it was more feasible to apply the unit load cost optimization method were observed as the initial unit load was designed with less stiff pallet top-deck boards; either thinner top deck boards or lower density wood species. For box-related variables, unit loads of smaller-sized boxes, unit loads with a smaller range of board grade reduction, and unit loads with higher initial board grades all created more favorable situations on which to apply the unit load optimization method due to the heavier weight of these unit loads.

## 4. Limitations and Assumptions

1. Only a standard GMA™ style, stringer class, wooden pallet design was investigated in the study.
2. PDS can only run analysis up to 38.1 mm top deck board thickness, so the scenarios requiring top deck boards thicker than 38.1 mm were not simulated. Therefore, the color grading system was applied to show the comparison between these different scenarios.
3. Due to the functional limitations of PDS™ regarding top deck thickness increases, the correlation between pallet stiffness (kg/mm top deck deflection) and the amount of wood material that needed to be added was not investigated.

## 5. Conclusions

The key findings of this study were as follows:

- The benefits of increasing a pallet’s top deck thickness to reduce the corrugated board grade diminish as the initial stiffness of the pallet increases.
- There were more opportunities to optimize unit load designs when the ECT values between the different board grade levels were lower.
- There were more possibilities of decreasing board grade when the initial board grade was higher, and/or when the box size was smaller, mostly due to the heavier weight of these unit loads, which caused greater pallet bending. Pallets made of Kiln-Dried Southern Yellow Pine are less likely to be able to be optimized using the investigated methods because of the limited deck board thicknesses that can be cost-effectively manufactured from the available raw materials.

Therefore, this study suggests that companies that use low stiffness pallets or have unit loads of heavy boxes could have more opportunities to optimize their unit loads by increasing the top deck thickness of their pallets.

The study also revealed that changing the top deck board stiffness cannot be done without considering the effects of other factors such as initial top deck board thickness, pallet wood species, box size, and board grade. Therefore, the unit load optimization process that reduces corrugated board grade by increasing top deck stiffness needs to be a holistic process.

The next phase of the project will focus on investigating whether the increase in pallet top-deck stiffness and the resulting reduction in corrugated boxboard grade can create an environmentally beneficial scenario.

## Figures and Tables

**Table 1:** Summary table of variable factors related to pallets.

**Pallet wood species**	Green high-density hardwood
	Green low-density hardwood
	Green southern yellow pine
	Kiln-dried southern yellow pine
**Initial top deck thickness for green lumber**	9.5 mm
	12.7 mm
	15.9 mm
	19.1 mm
**Fixed range for KD SYP lumber thickness**	11.1 mm to 17.5 mm

**Table 2:** Summary table of variable factors related to corrugated boxes.

Box Size (mm)	Flute Size	ECT Range (kg/mm)
Small (203.2 × 304.8 × 254)	C	0.57 to 0.52
		0.71 to 0.57
Medium (406.4 × 254 × 254)		0.79 to 0.71
	BC	0.91 to 0.86
Large (609.6 × 337.8 × 254)		1.09 to 0.91
		1.27 to 1.09

**Table 3:** Summary table of box load and compression strength factors.

	Box Load and Compression Strength Factor							
	Small C-Flute Box		Large C-Flute Box		Small B-Flute Box		Large B-Flute Box	
Topdeck Thickness (mm)	PDS	Experiment	PDS	Experiment	PDS	Experiment	PDS	Experiment
9.5	1.366	1.362	1.332	1.363	1.375	1.513	1.360	1.334
12.7	1.268	1.145	1.225	1.152	1.307	1.320	1.258	1.172
15.9	1.187	1.090	1.153	1.116	1.222	1.298	1.179	1.117
19.1	1.136	1.005	1.109	1.075	1.165	1.105	1.129	1.022

**Table 4:** The amount of top deck board thickness required to optimize unit loads consisting of C-flute boxes.

			Amount of Top Deck Thickness Increase (mm)								
		C-Flute	0.57–0.52 kg/mm ECT			0.71–0.57 kg/mm ECT			0.79–0.71 kg/mm ECT		
		Initial Top Deck Thickness (mm)	Grn HD HW	Grn LD HW	Grn SYP	Grn HD HW	Grn LD HW	Grn SYP	Grn HD HW	Grn LD HW	Grn SYP
C-Flute	Small	9.5	3.2	3.2	3.2	8	6.4	8	3.2	3.2	3.2
		12.7	4.8	4.8	6.4	N/A	N/A	N/A	4.8	3.2	4.8
		15.9	15.9	9.5	N/A	N/A	N/A	N/A	9.5	6.4	9.5
		19.1	N/A	N/A	N/A	N/A	N/A	N/A	N/A	N/A	N/A
	Medium	9.5	4.8	4.8	4.8	22.2	22.2	12.7	3.2	3.2	3.2
		12.7	12.7	8	8	N/A	N/A	N/A	8	6.4	4.8
		15.9	N/A	N/A	N/A	N/A	N/A	N/A	N/A	N/A	15.9
		19.1	N/A	N/A	N/A	N/A	N/A	N/A	N/A	N/A	N/A
	Large	9.5	8	9.5	9.5	N/A	N/A	N/A	9.5	11.1	11.1
		12.7	N/A	19.1	19.1	N/A	N/A	N/A	12.7	12.7	12.7
		15.9	N/A	N/A	N/A	N/A	N/A	N/A	N/A	N/A	N/A
		19.1	N/A	N/A	N/A	N/A	N/A	N/A	N/A	N/A	N/A

Grade 1: less than 12.7 mm (green), Grade 2: 12.7 mm to 25.4 mm (yellow), Grade 3: beyond 25.4 mm increase (red). Note: Grn HD HW: green high-density hardwood, Grn LD HW: green low-density hardwood, Grn SYP: green southern yellow pine.

**Table 5:** The amount of top deck board thickness required to optimize unit loads consisting of BC-flute boxes.

			Amount of Top Deck Thickness Increase (mm)								
			0.91–0.86 kg/mm ECT			1.09–0.91 kg/mm ECT			1.27–1.09 kg/mm ECT		
		Initial Top Deck Thickness (mm)	Grn HD HW	Grn LD HW	Grn SYP	Grn HD HW	Grn LD HW	Grn SYP	Grn HD HW	Grn LD HW	Grn SYP
BC-Flute	Small	9.5	1.6	1.6	1.6	4.8	4.8	4.8	4.8	4.8	4.8
		12.7	3.2	1.6	1.6	19.1	6.4	6.4	8	6.4	6.4
		15.9	3.2	3.2	3.2	N/A	N/A	N/A	N/A	N/A	15.9
		19.1	12.7	3.2	6.4	N/A	N/A	N/A	N/A	N/A	N/A
	Medium	9.5	3.2	3.2	1.6	8	6.4	6.4	6.4	6.4	6.4
		12.7	3.2	3.2	3.2	N/A	19.1	19.1	12.7	9.5	8
		15.9	6.4	4.8	4.8	N/A	N/A	N/A	N/A	N/A	N/A
		19.1	N/A	12.7	12.7	N/A	N/A	N/A	N/A	N/A	N/A
	Large	9.5	6.4	9.5	8	N/A	N/A	N/A	N/A	N/A	N/A
		12.7	6.4	6.4	6.4	N/A	N/A	N/A	N/A	N/A	N/A
		15.9	9.5	9.5	9.5	N/A	N/A	N/A	N/A	N/A	N/A
		19.1	N/A	N/A	N/A	N/A	N/A	N/A	N/A	N/A	N/A

Grade 1: less than 12.7 mm (green), Grade 2: 12.7 mm to 25.4 mm (yellow), Grade 3: beyond 25.4 mm increase (red). Note: Grn HD HW: green high-density hardwood, Grn LD HW: green low-density hardwood, Grn SYP: green southern yellow pine.

**Table 6:** The amount of top deck board thickness required to optimize unit loads consisting of KD SYP pallet and C-flute boxes.

			Amount of Top Deck Thickness Increase (mm)		
			0.79–0.71 kg/mm ECT	0.71–0.57 kg/mm ECT	0.57–0.52 kg/mm ECT
		Initial Top Deck Thickness (mm)	Kiln-Dried Southern Yellow Pine		
C-Flute	Small	11.1	17.5	N/A	17.5
	Medium		N/A	N/A	N/A
	Large		N/A	N/A	N/A

Note: The deckboard thickness sizes available for kiln-dried southern yellow pine (KD SYP) were limited because the available raw material size only allows the cost-effective production of 11.1 mm and 17.5 mm deckboard thicknesses.

**Table 7:** The amount of top deck board thickness required to optimize unit loads consisting of KD SYP pallet and BC-flute boxes.

			Amount of Top Deck Thickness Increase (mm)		
			0.91–0.86 kg/mm ECT	1.09–0.91 kg/mm ECT	1.27–1.09 kg/mm ECT
		Initial Top Deck Thickness (mm)	Kiln-Dried Southern Yellow Pine		
BC-Flute	Small	11.1	N/A	N/A	17.5
	Medium		N/A	N/A	17.5
	Large		N/A	N/A	17.5

Note: The deckboard thickness sizes available for kiln-dried southern yellow pine (KD SYP) were limited because the available raw material size only allows the cost-effective production of 11.1 mm and 17.5 mm deckboard thicknesses.

## Data Availability

The data presented in this study are available on request from the corresponding author.
